# The Citizen and the Public Health---the Individual’s Relation to the Health of the Community*An address delivered at the National Conservation Exposition, Knoxville, Tenn., on Public Health Day, October 25, 1913. Reprint from Public Health Reports, Vol. XXVIII, No. 45, November 7, 1913.

**Published:** 1914-02

**Authors:** John W. Trask

**Affiliations:** Assistant Surgeon General, United States Public Health Service


### Abstracts and Selections.

### The Citizen and the Public Health---The Individual's Relation to the Health of the Community.*

BY JOHN W. TRASK, ASSISTANT SURGEON GENERAL, UNITED STATES PUBLIC HEALTH SERVICE.

There are few things of so great importance to the individual as his health. Upon it depends largely his attitude toward life and his relationship to his fellow man. Generall speaking, those physically well are prosperous and efficient and the sick or diseased unsuccessful and inefficient.

The individual chronically poisoned by malaria or by hookworm infection finds his daily work onerous and the fruits of his labor give but little pleasure. The consumptive would gladly exchange his bank account for physical health. Who would not give his material wealth if by so doing he could bring back loved ones lost prematurely by fatal disease?

The health of the community is the combined health of those living in it. The relation of the citizen to the health of the community is therefore his relation to the health of his neighbors and of those living in the same city or State.

The health of the community should be of interest to every individual, for upon it depends the welfare of himself, of his family,

*An address delivered at the National Conservation Exposition, Knoxville, Tenn., on Public Health Day, October 25, 1913. Reprint from Public Health Reports, Vol. XXVIII, No. 45, November 7, 1913.

and of his fellow citizens. Upon the health of the people depends the happiness and prosperity of the community. Without health there can be no real prosperity and such material success as may be attained is of little benefit.

To the extent that the inhabitants of a community are sick the community itself is diseased. The community has health only in so far as the people are free from disease. To a community health is a valuable asset. It insures prosperity. It attracts people. It increases the value of the land. Many letters are received daily at the Public Health Bureau at Washington from people who are contemplating buying land or moving from one State to another asking about the health conditions of certain localities. They want to know whether there is much sickness in this or that locality, whether there is any malaria, much typhoid fever or tuberculosis, and whether there is a pure water supply. People are thinking in these days of their physical welfare, and have no desire to live in localities where insufficient attention is given to the prevention of disease and where there is more sickness than there should be. The community that has health has a distinct advantage in the competition for economic prosperity over the sick community.

The health of the community depends upon the health of the citizens, but the health of each individual also depends in some measure, often in large measure, upon that of the other members of the community. Health of the individual is therefore a condition that, generally speaking, can be maintained only by a combination of individual and community effort, and its importance is such that in the activities of the city and of the State it should hold a prominent place. The health of the community should be of greater concern than commercial prosperity, for it is essential to commercial prosperity. Necessary as are our courts, our fire and police departments, and our educational systems, the importance of the community's attention to the citizen's health is second to none.

Each case of a communicable disease in a city threatens the welfare of every citizen. Every case of tuberculosis or of typhoid fever is to some degree a menace to every uninfected person. Modern civilization in its development has become more complex, and as a result of the many avenues of social and commercial intercourse we are brought more frequently into contact with our fellow man and his life.

Where the bread is baked in the home people are not exposed to the diseases of the bakers and of those who handle the bread in shops, but in cities most bread is not baked in the home. Today a number of cities properly require that no person afflicted with any communicable disease shall be employed in a bakeshop, and that bread and other articles made in bakeries shall be wrapped in paper before leaving the bake room. More than one State now has regulations requiring the wrapping of bread in this way throughout the State.

If we patronize barber shops, we are liable to be exposed to certain diseases of the many patrons who have preceded us unless special precautions are taken. We are likewise exposed to the diseases of our servants, and not only to their diseases, but to the diseases in the families and houses from which they come. We may be exposed to the diseases of those who send their clothes to the same laundry in which our clothes are washed unless there are proper supervision and regulation.

When there is a family cow or a cow supplying a small neighborhood the possibility of the milk carrying disease is comparatively limited. But in cities where milk dealers receive their milk, often from hundreds of farms, and after mixing it in large tanks distribute it to thousands of people, the danger from chance contamination of the milk with disease germs is many times greater, for instead of one family handling the milk there may be hundreds, and if the milk from any one farm is infected with typhoid or scarlet fever germs all the milk may become contaminated when it is mixed in the vat of the city distributor and hundreds of families thus exposed to infection. This is not merely problematical. It is a thing of frequent occurrence. Epidemics of typhoid fever due to infected milk are common. Outbreaks in which there have been many hundreds of cases of scarlet fever or diphtheria have been caused by milk in a number of cities. Large outbreaks of septic sore throat, spread by milk, have within the last two years occurred in Baltimore, Boston, Chicago, and elsewhere.

In street cars we come into close contact with people from many homes. And there are still other means by which we are brought into contact with our fellow citizens and their diseases. The fly that breeds in garbage, decaying vegetation, and stable refuse and feeds on anything and everything, including the sputum of consumptives, the excretions of typhoid patients, and the pus discharged from sore eyes and running ears, by its sociable habit of going from one house to another may carry diseases to people who never see the sick.

At church we come into more or less close contact with people from many houses, in some of which there may be persons sick with communicable diseases. At day school and in Sunday school children are associated with others and frequently contract disease, as is well known to all. The diseases of children are often spread in this way. It is only proper, therefore, that each household give special attention to the welfare of other households by keeping at home those sick with communicable diseases until all danger of spreading the diseases is past. This is especially true of the acute infectious diseases, such as measles, scarlet fever and diphtheria. When these are known to be present in the community, parents should be watchful, for frequently children are sick for some time before the nature of the illness is recognized, and, if during this time they mingle with others, the disease is likely to be spread, and no right-minded citizen wishes by lack of due care to be responsible for the occurrence of sickness in others, sickness that may deprive others of life.

The common drinking cup, which until recently it was customary to see at drinking fountains and in public places, brought individuals into almost personal contact. Every person who drank left a little of his saliva and a few of the germs from his mouth on the edge of the cup, and in using the cup not only quenched his thirst but sampled, as it were, the salivary contributions and the germs of his predecessors.

What is true of the common drinking cup is likewise true in some measure of cups, glasses, spoons, and forks in restaurants, hotels, and at soda water fountains, if they are not properly cleansed after being used. The possible danger in placing to our mouths cups or other vessels that have been used by persons of whose conditions of health we do not know will be readily appreciated if we consider tuberculosis. This disease is present throughout the world. About one person in every hundred in our cities has it in a form in which it may be spread to others. A small proportion of the cases in man is contracted from milk from tuberculous cows. This is especially true of the disease in children. With the exception of this comparatively small proportion, the disease is spread from person to person, and each afflicted individual owes his misfortune to the fact that he either unduly exposed himself or was not properly protected from the disease in some one else. As the germ which causes tuberculosis is usually present in sputum and mouths of consumptives, the possible danger in using a common cup of any kind is readily apparent.

The common towel and the common comb and brush of the waiting room or other public place all contribute to bring their users into very close relationship, a relationship usually closer and more intimate than that of ordinary social intercourse with friends and acquaintances.

Do what we will, our health depends not only on how we live but also on how the other people of the community live. The danger of infection from the sick and diseased we never see is often greater than that from the sick we see. We can protect ourselves from those we see and know of, but we are in large measure helpless to protect ourselves from those of whose existence we are unaware.

Every case of a communicable disease in a city is directly or indirectly a menace to every person. The safety of every inhabitant depends upon the health of the community.

In the complex life of modern civilization we cannot individually protect ourselves from infection, so to prevent the spread of disease from the many sick we do not see and seldom know exist and to watch and control the common avenues by which disease is usually communicated, we employ for the community a health officer and give him a certain number of assistants to help him and an appropriation of money for the expenses of his work.

The health department is a department created and supported by the people to look after the community's health, to protect them and their neighbors from unnecessary exposure to sickness. The health department is your department, doing the things for you that you cannot do for yourself, and, being the creature of the community, the community's servant, as it were, the health department will be as efficient and as watchful as the people insist that it shall be or allow it to be. It cannot be more so.

The health department and its work represents the desire of the people to avoid disease, to live useful wholesome lives, to protect themselves, their children, and their families. It represents not only the self-interest of the individuals, but their altruism as well. It represents one of the finest products of our civilization, the realization that health is the right of every man and that the preservation of one's own health and that of his neighbor is a moral duty.

It is the result of knowledge that disease is not a necessary evil sent by a chastising God, but is caused either by living things we call germs, which we get by direct or indirect contact with the sick or by improper living.

The health department is the result of our knowledge that disease can be prevented and that the degree of the community's health depends upon the desire of the citizens to have health, their intelligence and the amount of effort they are willing to make individually and through their municipal or other government to attain it.

The work of the health department should mean more to the community than the perfunctory performance of certain duties. It should be a thing of vital interest to every individual, and as such should receive his earnest co-operation.

A part of the work of every health department is the enforcement of the laws and regulations which the people have had adopted for the protection of the community's health. Every intelligent citizen should know what these laws and regulations are. He should also compare them with the laws and regulations of other communities, that he may know whether his city or State is doing as much as it should to protect a thing of so great importance to the individual and general welfare as the community's health.

But of more importance than the enactment of laws or the promulgation of regulations is their enforcement. It is not the laws on the statute books that are of value, but the ones that are enforced.

Every thoughtful citizen should know what work the health department is doing and the extent of protection from disease that is being given to him and to those dear to him. Such interest will in itself insure more efficient work, for the health department needs the interest of all intelligent citizens. It needs their moral support, their approval of work accomplished and at times their co-operation.

Every household should see that it does not spread disease to others, that it does not become a focus of infection endangering the welfare of the community.

Every citizen should keep his premises clean; should see that he is not maintaining collections of garbage or refuse in which flies may breed. He should see that all sanitary regulations are complied with, and then should supplement these with as many more as his knowledge tells him will be useful. Whenever any member of his household contracts a communicable disease, he should take such precautions as will prevent its being spread to others. He should bear in mind that every case of a communicable disease is contracted directly or indirectly from some infected person, and that the case in his family is probably due to some one's neglect of his responsibilities to the community. His household should not become the cause of the further spread of the diseasee. If the disease is one that should be reported to the health department, he should see that this is done, and in any case if in doubt he should communicate with the health department for advice or instructions, for the health department is maintained by him and his fellow citizens for this purpose.

But the health of the community relates to more than the matters we have been discussing. It includes more than the prevention and control of the communicable diseases. It includes the prevention of the diseases due to improper living, to improper working conditions, and to faulty construction and management of schools. The health of the community is intimately connected with all its activities, social and economic. The hours and conditions of labor in the industries, the pay of employes, the price of land and the construction of dwellings, especially for those whose incomes are small; all have an important bearing on the health of the people.

In all of these the citizen should be interested both from altruistic motives and because the preservation of health pays, and is to the economic advantage of the individual, the employer of labor, and the community as a whole.

The citizen will be interested in the community's health only when he understands its significance and importance. The advancement of the health of the community, therefore, depends in large measure on bringing to the attention of the people the causes of disease and the possibility of their prevention. In doing this the schools can be useful. Children can be taught the essentials of hygiene and sanitation. What can a child learn in school of greater importance than how to keep well and strong? Then, too, the churches can do their part. As leaders in the better tendencies of humanity and in most of our altruistic activities the church has a rare opportunity to aid in the promotion of health work. Another factor which must not be overlooked, for it has proved to be one of the most powerful influences for sanitary betterment in many localities, is the women of the community. Women, when organized and working together, have shown themselves to be one of the most potent factors in awakening the community conscience to the importance of the protection of health and the maintaining of orderly, wholesome cities.

When the people in a community desire sufficiently to have good schools, or good roads, or efficient police protection, they have no difficulty in getting them. When they desire health protection and the prevention of disease, these are as readily obtainable.

